# Portable spectroscopy, digital imaging colorimetry and multivariate statistical tools in contaminant identification: A case study of mint (*Mentha*) and basil (*Ocimum basilicum*)

**DOI:** 10.1016/j.heliyon.2024.e30924

**Published:** 2024-05-17

**Authors:** Zina-Sabrina Duma, Tuomas Sihvonen, Erik Vartiainen, Satu-Pia Reinikainen

**Affiliations:** LUT University, Yliopistonkatu 34, Lappeenranta 53850, Finland

**Keywords:** Handheld instrumentation, Raman spectroscopy, Fourier-transform infrared (FTIR) spectroscopy, Colorimetry, Plant contamination

## Abstract

The advent of portable Fourier-Transform Infrared (FTIR) and Raman spectrometers has revolutionized analysis capabilities, presenting the possibility of on-site contaminant identification without the need for specialized laboratory settings. Compared to laboratory instrumentation, portable spectroscopy is more prone to noise, and appropriate spectral processing procedures need to be established. This paper introduces a comprehensive methodology that integrates acquisition techniques, spectral analysis, and mathematical tools necessary for utilizing handheld spectrometers to diagnose plant contamination. It focuses on determining the efficacy of handheld FTIR, Raman spectroscopy, and digital imaging for detecting contaminants in two food plants, Basil (*Ocimum basilicum*) and Mint (*Mentha*). The study examines the impact of three pollutants: iron (II) sulphate ($FeSO_{4}$), zinc (II) sulphate ($ZnSO_{4}$), and copper (II) sulphate ($CuSO_{4}$), on these plants, but also the necessary amount of measurements to spot the pollutants' effects. Measurements were conducted at the start, after 24 hours, and after 48 hours of exposure, on both fresh and dried plant leaves, as well as in solution. Spectral effects of each of the pollutants were identified with the use of multivariate statistical process control techniques. With the help of the developed methodologies, researchers can identify *in-situ* contaminant effects, exposure times and run diagnostics directly on the leaf both in alive and dried plants.

## Highlights

• Handheld instrumentation gives noisier spectra than laboratory instrumentation.
• Appropriate mathematical handling makes handheld data usable.
• Colorimetric studies can be utilized for plant control.
• $Cu^{2+}$, $Zn^{2+}$ and $Fe^{2+}$ contaminants are detected in plants with handheld instrumentation.

## 1 Introduction

As climate change progresses, monitoring plants has become increasingly crucial. Both natural and agricultural plants are affected by anthropogenic factors, necessitating accurate diagnostics to prevent adverse outcomes. Fourier-transform infrared spectroscopy (FTIR) is widely used for plant monitoring due to its non-destructive nature, preserving the integrity of the plants being studied [1], [2], [3], [4], [5]. Similarly, Raman spectroscopy has gained recognition for its effectiveness in diagnosing plant diseases and pollutants [6], [7], [8], [9], [10].

Portable instruments enable on-site analysis in agricultural or natural settings, eliminating sample disturbances caused by transportation to a laboratory. Although handheld devices are still developing, their potential has sparked significant interest [11], [12], [13].

Most of the present literature showcases spectroscopical methods to identify diseases on plant leaves instead of plant contamination or pollutant identification. The traditional way to identify plant diseases is in a laboratory setup, through DNA and serological methods, along with methods based on nucleic acid and protein analysis [14]. Spectroscopical methods are becoming more popular in recent years. In a comprehensive review, Fang et al., 2023 [15] presents applications of multiple spectral systems for tree disease detection. Amongst the mentioned methodologies are handheld Raman spectrometers and multispectral Unmanned Aerial Vehicles (UAVs). Another review by Weng et al., 2021 [7] adds the Surface-Enhanced Raman Spectroscopy (SERS) to the aforementioned list. Faber et al., 2019 [16], explores, in addition, the Polymerase Chain Reaction (PCR) and Quantitative Polymerase Chain Reaction (qPCR), Enzyme-Linked ImmunoSorbent Assay (ELISA), Fluorescence In-Situ Hybridization (FISH), ImmunoFluorescence Imaging (IFI), Flow Cytometry (FCM), Thermography, RGB and Fluorescence Imaging.

Amongst the studies reviewed, the classification of healthy and diseased plants was made through K-Kearest Neighbours (KNN), Logistic Regression (LR), Support Vector Machines (SVM), Finite Difference Approximation (FDA), Bivariate Correlation (BC), Partial Least-Squares with Discriminant Analysis (PLS-DA), Multilayer Perceptron (MLP), Naïve Bayes Classification (NBC) and Random Forest (RF). While the methods are efficient in discriminating between a healthy state and a known disease, data is needed on all disease states for disease identification. Another drawback of the aforementioned methods is that, apart from PLS-DA, the methods are not interpretable, and additional analysis is needed to identify the wavenumbers or variables that change with the evolution of the disease.

While tree diseases are identified with leaf spectra, there is a gap in the literature for analyzing thin, food plant contaminants such as those of *Basil* and *Mint*. This research aims to answer key questions:

• *Can portable Raman and FTIR instruments identify pollutants affecting live food plants?*
• *Do these instruments provide insights into a plant's pollutant exposure duration?*
• *Is there detectable leakage from plants within the limits of a portable Raman instrument?*
• *Can portable FTIR instruments identify the causative factors after a plant's death?*
• *Can widely available digital imaging be utilized in plant contamination?*

Experiments were conducted on mint (*Mentha*) and basil (*Ocimum basilicum*), two plants of agricultural significance. While basil has been studied under physical and chemical stresses, Raman spectroscopy in these studies focused on derived oils, not directly on the plant leaves [17], [18], [19], [20]. In contrast, mint plant analysis using Raman spectroscopy has primarily dealt with chemotaxonomy [21] and harvest time determination [22], often analyzing processed mint oil.

The plants were exposed to three metal pollutants: iron (Fe), zinc (Zn), and copper (Cu), which are among the eight essential micronutrients for plants [23], along with boron (B), chlorine (Cl), manganese (Mn), molybdenum (Mo) and nickel (Ni). The elements were chosen as surrogate metal contaminants to demonstrate the feasibility of the method. FTIR and Raman spectroscopy measurements were taken from healthy and exposed plants 24 and 48 hours after the beginning of the exposure. FTIR analyzed dried plants, while Raman spectra were obtained from live leaves and the immersion solution. Control samples were also monitored at the same intervals.

The mathematical methods used to model the spectra are based on multivariate statistics. To ensure the measurements reflect variation related to the biochemical properties of the plants, instrumental and interference noise is excluded by smoothing and baseline-correcting the spectra [24]. The baseline correction can be automatic or manual. In manual baseline correction, one needs to select baseline points to be aligned [25]. The automatic baseline estimation algorithms include asymmetric least-squares, robust baseline estimation, wavelets, iterative polynomial fitting and rolling balls [26]. Segments of importance from the treated spectra are modelled using Principal Component Analysis (PCA) [27]. Investigative tools derived from Multivariate Statistical Process Control (MSPC) [28], such as prediction error control charts and Hotelling's $T^{2}$ control charts, are constructed to spot unusual variation induced by the pollutants to the leaf spectra.

The novelty of this research lies in its use of portable instruments for direct leaf measurements of food plants, development of experimental and mathematical procedures for plant health monitoring using portable Raman and FTIR spectra, incorporation of process control charts, and the addition of digital imaging in combined-method plant health assessment models. The importance of the study to the research community stands in a complete methodology plant-to-diagnostic, introducing variograms to estimate appropriate sample sizes and multivariate statistical process control charts for health state monitoring.

Compared to the classification-based models reviewed in the literature, the present study only needs healthy plants for model calibration and can identify chemical stressors of the plant regardless of their nature. There is no need for separate models for each of the pollutants. Classification-based models work only for the disease scenarios they have been trained on, whereas multivariate statistical control charts can identify any deviation from the healthy state of the plant. Classification-based methods also need separate tools to identify specific spectral changes, as many of the methods are closed, with no insights into variable importance (e.g. KNN, MLP, SVM). In the proposed scenario of the present case study, the contributions of wavenumbers to control charts give the influence of variables in detecting plant changes.

## 2 Experimental methods

The present section presents the experimental design in Subsection 2.1, along with the instrumental descriptions for FTIR spectroscopy (Subsection 2.2) and Raman spectroscopy (Subsection 2.3). Digital Imaging is presented in Subsection 2.4.

### 2.1 Experimental design and sample preparation

**Figure 1:**
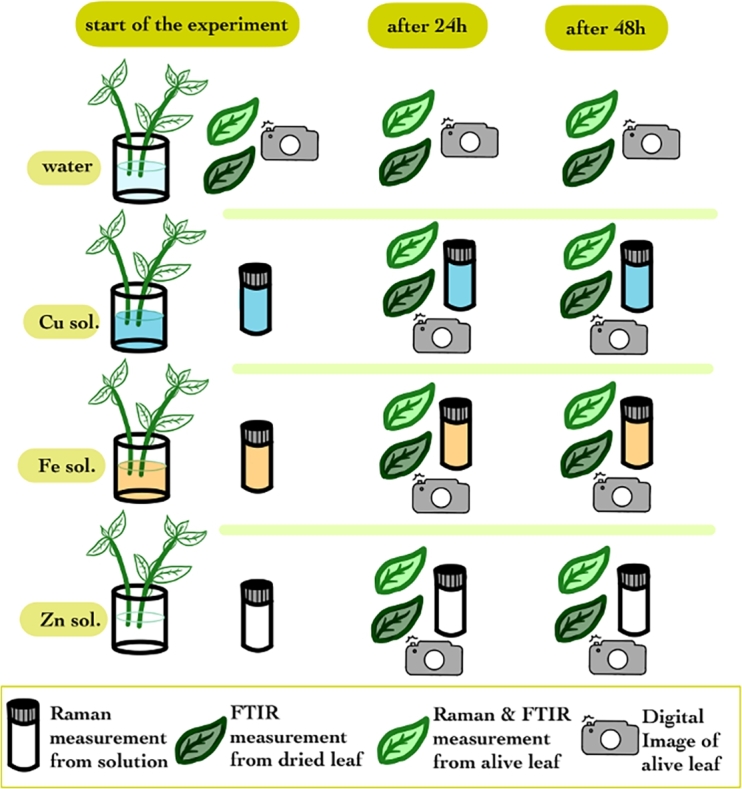
Experimental design for mint and basil plants. The solution is analyzed with the Raman instrument, the live plant with Raman, FTIR and digital imaging, and the dried plant is analyzed with the FTIR spectrometer.

The experimental procedure for both mint and basil plants was identical. Fig. 1 illustrates the experimental process. Initial measurements were taken using Raman and FTIR spectrometers for the solutions and fresh leaves. Additionally, dried leaves from the initial batch of plants were analyzed with the handheld FTIR spectrometer. Digital images were taken initially and at each experimental breakpoint. This measurement protocol was consistently followed for subsequent assessments at the 24-hour and 48-hour marks. The samples were dried in air for two weeks on a glass support. No thermal treatment has been applied to fasten the drying to the leaves to mimic the natural drying.

Determining the necessary number of samples to calibrate the control charts is done using a variogram and sample batch standard deviation. The total number of samples considered for variogram analysis was 60 for both basil and mint, taken from different parts of the leaf. Because the leaf surface is not homogeneous, the PCA model should contain enough samples that include representative variation of the plant, including young and old leaves and various points on the leaf surface. After the healthy-plant PCA model has been calibrated with the number of samples revealed by the variogram, the number of polluted plant samples to be projected onto the model is not limited. We have chosen a number of 10 samples to be taken after each experimental checkpoint, both from control and polluted plants, as the effects of the pollutant can differ from leaf to leaf and also between different areas on a leaf.

The focal point of the Raman spectrometer cannot be precisely determined when acquiring measurements. In the FTIR case, the evanescent wave of the FTIR could penetrate through the leaf and acquire spectral information related to the background if only one leaf is analyzed at a time. For the above-mentioned reasons, the alive leaves were taken into stacks of leaves as opposed to a single leaf under the instrument, and their order was rotated throughout the measurements, in a random stratified sampling strategy. The fragile dried leaves are crushed under the weight of the instrument, and a homogeneous layer of powder-like leaves is analysed. Between each measurement, the dried leaf particles are mixed, resulting in a homogenized random sampling layer.

Table 1 details the concentrations used in the solutions. In each instance, a sulphate salt of the respective metal was dissolved in water. Notably, the mint plants were smaller in size compared to the basil plants and thus were placed in solutions with reduced volumes. However, the molar concentrations of the solutions were maintained consistently across both plant types. All salts are of laboratory purity and meet analytical specification: Copper(II) sulphate, $CuSO_{4}$ (WWR Chemicals, purity >99%), iron (II) sulphate sesquihydrate, $FeSO_{4}1.5H_{2}O$ (Riedel-de Haen, Sigma-Aldrich, purity >98%), zinc (II) sulphate heptahydrate, $ZnSO_{4}7H_{2}O$, (Fluka, Sigma-Aldrich, purity >99%).

**Table 1:** Molar concentrations of the solutions the plants are immersed in.

Plant	Pollutant	Water [L]	Mass salt [g]	Concentration [mol/L]
Basil	*CuSO*_4_	0.2	0.2	0.00627
	*FeSO*_4_ 1.5*H*_2_*O*			0.00556
	*ZnSO*_4_ 7*H*_2_*O*			0.00348

Mint	*CuSO*_4_	0.08	0.08	0.00627
	*FeSO*_4_ 1.5*H*_2_*O*			0.00556
	*ZnSO*_4_ 7*H*_2_*O*			0.00348

### 2.2 FTIR spectroscopy

**Figure 2:**
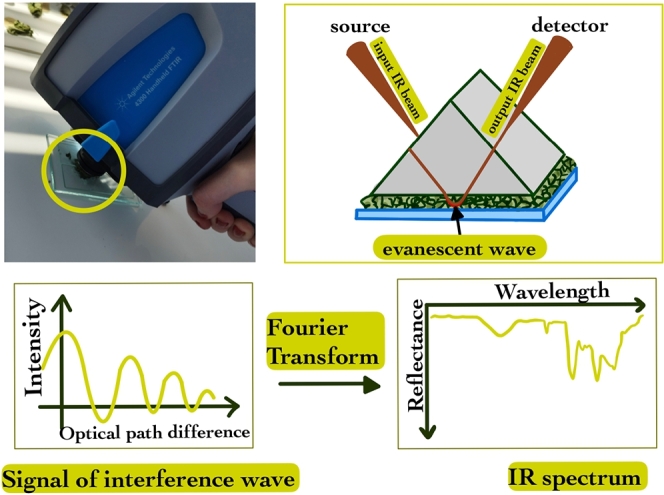
Principles of handheld FTIR instrumentation and spectral acquisition.

Handheld FTIR spectroscopy is gaining popularity for its cost-efficiency in chemically identifying plants [29]. It employs Attenuated Total Reflection (ATR) technology, as shown in Fig. 2. In the present study, a diamond (D-ATR) medium is utilized. Depending on the absorbed light on the sample, a different quantity of IR light exits the medium and is counted at the detector. The signal acquired by the FTIR spectrometer is seen in Fig. 2 and is called an interferogram. With the increased optical path difference, separate wavenumbers produce peaks at different positions. The frequency of the signal is inversely proportional to its wavenumber [30].

The portable FTIR spectrometer utilized in the study is an Agilent 4300 Handheld FTIR spectrometer. The reflectance is acquired with the use of a diamond ATR probe, which analyzes the top 2 microns of the surface and has a focal spot size of 2 mm. The spectral acquisition respected the following steps: **Step I.** The diamond ATR is wiped with an isopropyl-damped cloth; **Step II.** Background spectra are collected; **Step III.** The contact with the sample is ensured, and pre-sample alignment checks and sample continuity checks are performed. **Step IV.** The actual spectra acquisition is made.

ATR correction is performed in the instrument that acquires spectra in ATR mode. The spectrum is collected in full range, from 4000 to 650 cm^−1^. The spectral resolution is 4 cm^−1^, with a scan speed of 2.4 Hz. For each spectral collection, there are collected 8 background spectra and 8 sample scans. The phase correction mode is *Mertz*, and the apodization is *Gapp-Genzel*.

### 2.3 Raman spectroscopy

**Figure 3:**
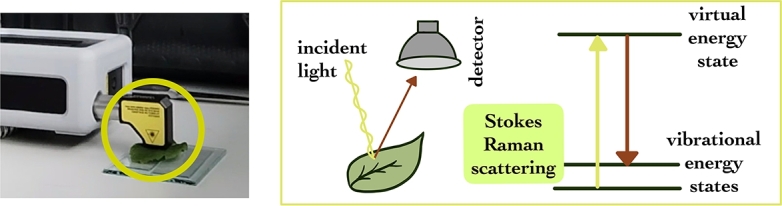
Principles of Raman spectroscopy and spectral acquisition using a right-angle cap.

In Raman spectroscopy, a sample is excited by a photon, usually from a laser. This excitation causes the electron of the sample molecule to be lifted to a higher energy state, as seen in Fig. 3. In Stokes Raman scattering, the electron returns to a higher vibrational state, and thus, there is a difference between the excitation and the emitted photon energies. This difference is recorded as the Raman shift [31].

The model of the Raman spectrometer utilized is TacticID-1064. The laser intensity is 378 mW for healthy green leaves and can be decreased in case of discolouring caused by pollutant-induced drying of the leaf. The instrument automatically determines the number of hits and the integration time, which depend on the Hit Quality Index (HQI), which is set to have a minimum value of 85. The average number of hits taken is 3, and the Raman shift range is between 176 and 2500 cm^−1^.

### 2.4 Digital imaging

As mentioned earlier, handheld spectrometry tends to be more susceptible to noise compared to bench-top spectrometry. This noise can lead to measurement heterogeneity. When such variability is observed, digital imaging analysis can help determine whether potential outliers are due to measurement noise or if certain leaf areas are more significantly affected by pollutants, resulting in divergent spectra.

**Figure 4:**
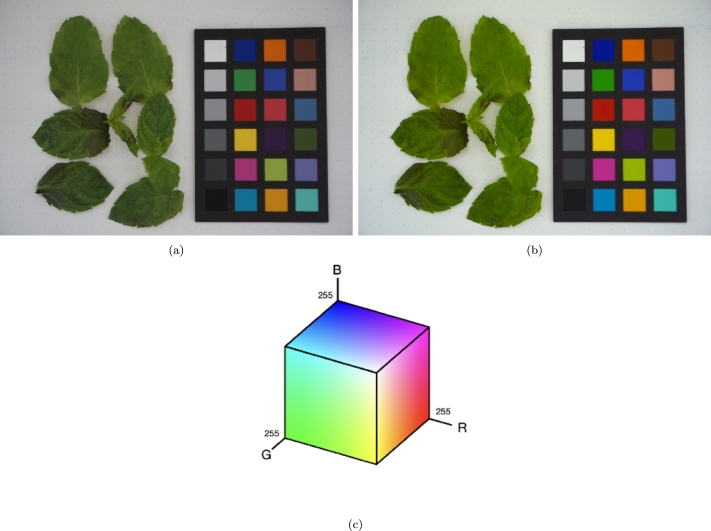
Digital image acquisition example for mint leaves 24 h *Cu*^2+^ exposure: (a) the raw digital image and the (b) color-corrected image. (c) Showcases the three-dimensional distribution of colours in the RGB colour space.

For consistent image analysis, maintaining uniform intensity across images is crucial. To achieve this, a ColorChecker board was placed adjacent to the leaves, and the images underwent colour correction following the methodology outlined by Duma et al., 2023. Examples of raw and colour-corrected leaf images are present in Figs. 4a - 4b. Post colour correction, the images were segmented to isolate individual leaves from each experiment.

The average colour of each leaf is represented as a point in a three-dimensional colour space, such as the RGB space shown in Fig. 4c. In a healthy plant, the average leaf colour should be consistent across leaves within the same experiment, leading to clustering in the colour space. Conversely, pollution effects can manifest as variations in the colourimetric distribution of the leaves within this space, indicating heterogeneity in surface colouration due to pollutant impact.

The camera type utilized for acquiring images in the present study is a Sony *α* 6000, with a 35 mm F1.8 lens. The acquisition mode was in regular *P* (Program), with ISO 100 automatic white balance, to result in high-quality JPEG mode. The image resolution is 24 MP.

The lighting conditions are not strict, as colour correction is performed, but it is important that there are no shadows on the leaf surface because it will falsely create heterogeneity on the surface. A diffuse light can be utilized to achieve illumination with no shadows. In the present study, the samples were placed in the shadow of natural lightning, ensuring no direct or secondary light at an angle creates shadows on the leaf surface.

## 3 Mathematical methods

The present section presents the theoretical mathematical approaches for solving spectral processing tasks such as baseline correction (Subsection 3.1), spectral smoothing (Subsection 3.2), PCA model calibration and control chart computation (Subsection 3.3). All mathematical methods for this study were implemented in MATLAB.

### 3.1 Baseline correction

The baseline appears due to the slow variation of the background during the acquisition of the spectrum and is variable from spectrum to spectrum for identical samples. The baseline can influence the interpretation of the spectra and alter the results of the multivariate analysis [32]. There are (i) manual and (ii) automated approaches to performing the baseline correction. The manual approach consists of selecting certain points on the spectrum and polynomial or spline interpolating the spectrum. The automatic methods rely on derivatives [33], morphological operators [34], wavelet decomposition [25], or iterative averaging [35]. In the present study, both iterative averaging methods and polynomial fitting are utilized to perform baseline correction. For Raman spectrum baseline correction, the automated MATLAB function can be found in the public repository of Al-Rumaithi, 2024 [36].

### 3.2 Spectral smoothing

A handheld instrument is more prone to noise from interference or background changes than bench-top instruments. The spectral smoothing step aims to de-noise the spectra and reveal the underlying peaks more clearly. The Savitzky-Golay filter is a common smoothing and de-noising technique applied to both Raman and FTIR spectroscopy [37]. Moving average filters involve replacing reflectance values by combining the signal values from a moving window centred at the wavenumber point. Savitzky-Golay fits a polynomial of a selected order through the reflectance in a wavenumber window and takes the central point of the polynomial curve to replace the reflectance value at the selected wavenumber [38].

### 3.3 PCA-based methodologies in pollutant diagnostics

Principal Component Analysis (PCA) is a critical chemometrics tool in spectral analysis, particularly valuable for handheld instrumentation known for noisier spectra [39] compared to bench-top alternatives. PCA distils systematic variations into Principal Components (PCs), allowing for the isolation of signal from noise [40], [41].

In PCA, noise-induced variations are minimized by truncating PCs predominantly influenced by noise, thus revealing the underlying, noise-filtered spectra. Given the chemical heterogeneity of plants, their spectral profiles (PCs) are expected to be varied and significant in contrast to samples of a single substance, where the first PC often explains most of the systematic variation in the measurements.

The inclusion of spectra from healthy plants in the model is guided by the method proposed by Paakkunainen et al., 2009, adapted for plant leaf spectra where variations may arise from leaf age and the spectral acquisition point, like the stem or leaf extremities.

**Figure 5:**
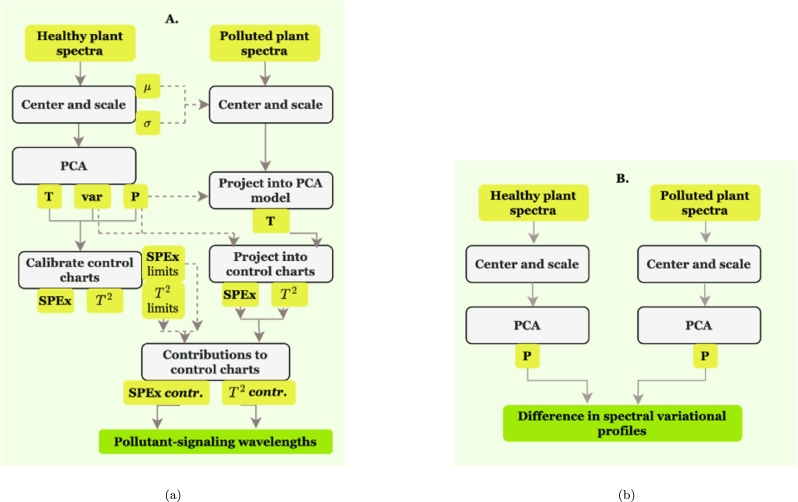
Workflows for identifying changing variation in wavenumber reflectance using Statistical Process Control methodologies.

PCA in this study was employed in two ways to identify pollutants or diseases in plants: firstly, by using a model of a healthy plant to identify deviations (**Scenario A**), and secondly, by comparing two distinct models for healthy and polluted plants (**Scenario B**). The methodologies for both approaches are delineated in Fig. 5.

In PCA decomposition, it is essential to standardize the spectra (**X**) to ensure each wavenumber contributes equally to the model, particularly when there's no specific information about the relative importance of different wavenumbers. The standardization process involves centring and scaling the data, as described in Eq. (1), where *μ* denotes the mean of each wavenumber and *σ* represents its standard deviation. In **Scenario A**, which involves projecting polluted data onto the model, standardization is achieved using the means and standard deviations from the calibration partition, which comprises spectra from healthy plants.

(1) $$\text{Z}=\frac{\text{X}-\mu }{\sigma }$$

Once centred, the data is projected onto Principal Component (PC) axes using either singular value decomposition or eigenvalue decomposition of the cross-product matrix. In this study, singular value decomposition was utilized. The spectra can be reconstructed with a truncated number of principal components. This process is demonstrated in Eq. (2), where **P** represents the loadings matrix, **T** denotes the scores matrix, and **E** refers to the residual matrix.

(2) $$\text{Z}=\text{T}{\text{P}}^{T}+\text{E}$$

The squared prediction error (SPEx) of an observation for a number n of PCs is calculated using (3), where $\overset{ˆ}{\text{Z}}$ is the reconstructed data for the PCs ($\overset{ˆ}{\text{Z}}=\text{T}{\text{P}}^{T}$) in Eq. (2) and, *i* is the wavenumber.

(3) $$SPE_{x}=\sum_{i=1}^{q}{\left(z_{i}-{\overset{ˆ}{z}}_{i}\right)}^{2}$$

Another control chart, based on Hotelling's $T^{2}$ score, is calculated using Eq. (4), where *t* is the score of the *j*-th PC, whereas *λ* is the variance if the *j*-th PC.

(4) $$T^{2}=\sum_{j=1}^{n}\frac{t_{j}^{2}}{\lambda_{j}}$$

If an observation has a high $T^{2}$ or SPEx value, the influence of individual wavenumber values on the indicators can be computed, giving a diagnostic for the polluted spectra [42]. To calculate the SPEx value contribution of a variable *i*, one can use Eq. (5).

(5) $$SPEx_{i,contr}={\left(z_{i}-{\overset{ˆ}{z}}_{i}\right)}^{2}$$

For calculating the variable contribution to Hotelling's $T^{2}$ value, Eq. (6) can be utilized.

(6) $$T_{i,contr}^{2}=\sum_{j=1}^{n}\frac{t_{j}}{\lambda_{j}}p_{i,j}(z_{i}-{\overset{ˆ}{z}}_{i})$$

## 4 Results and discussion

This section discusses the observable visual changes in plants subjected to pollutant stress alongside the impact of spectral pretreatment. It also covers the identification of polluted samples using statistical process control charts and the insights gained from PCA loadings.

### 4.1 Visual changes

**Figure 6:**
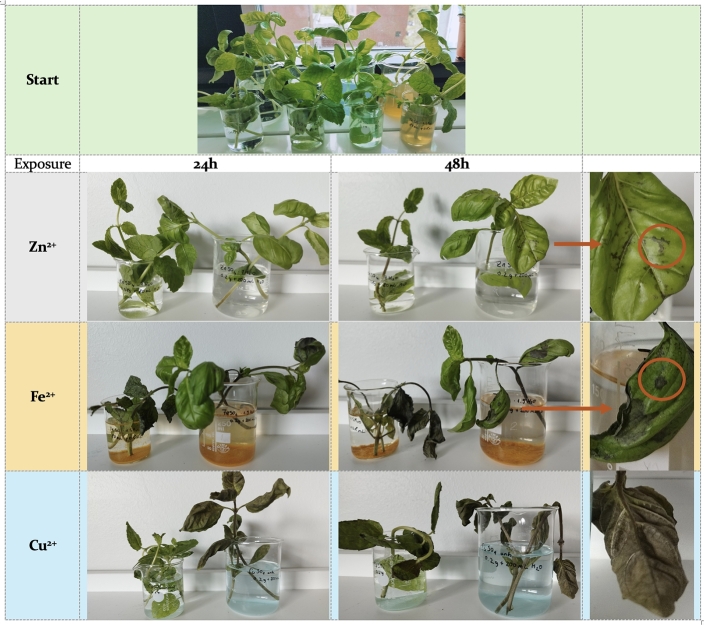
Visual changes of plants during experiments.

As depicted in Fig. 6, the visible effects of pollutants on plants intensify with time, becoming most pronounced after 48 hours. In the case of Zn, the salt utilized was a heptahydrate, and the overall $Zn^{2+}$ content in the solution is lower. It can be observed that dark spots appear on the leaf surface at 24 h, and they multiply in intensity and magnitude over time.

In the case of $Fe^{2+}$ exposure, the basil plant showed more susceptibility than the mint plant. The basil leaves rapidly turned brown and exhibited an increasingly dry texture after 48 hours of immersion. Conversely, the mint leaves developed a darker green hue, particularly at the extremities, eventually resulting in dark borders on all leaves after 48 hours.

The mint plant was significantly impacted by the $Fe^{2+}$ solution, becoming completely dry after 48 hours. On the other hand, the basil plant showed no immediate effects from the $Fe^{2+}$ solution within the first 24 hours. However, large dark spots emerged after 48 hours, leading to an overall drying of the plant.

### 4.2 Spectral pretreatment

**Figure 7:**
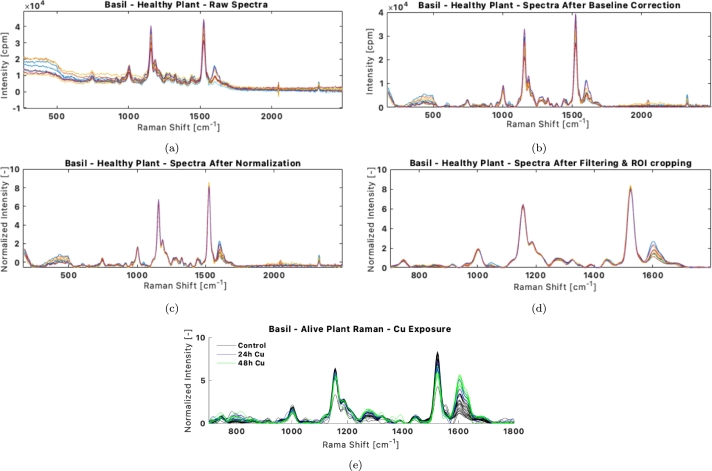
Example of pretreatment intermediate results for healthy Basil plant Raman observations: from the raw spectra (a), after the baseline correction (b), after normalization (c) and after smoothing and ROI-cropping (d). The spectra utilized in the PCA models are showcased in (d). (e) showcases the spectra for the control plants and the Copper exposure experiments.

Fig. 7 showcases an example of the results of spectral pretreatment applied to Raman spectra. Initially, Raman spectra exhibit fine noise and various background effects. A key step in pretreatment is baseline correction, performed here using an automated polynomial fit. Subsequently, the spectra are normalized spectral-wise, which helps align them for analysis. This normalization is crucial for the PCA model, as it minimizes false variations due to peak height differences across spectra, which are more likely caused by instrumental factors rather than differences intrinsic to the plant. Finally, the region of interest is extracted from the spectra, focusing on areas with the optimal signal-to-noise ratio and relevant spectral information.

**Figure 8:**
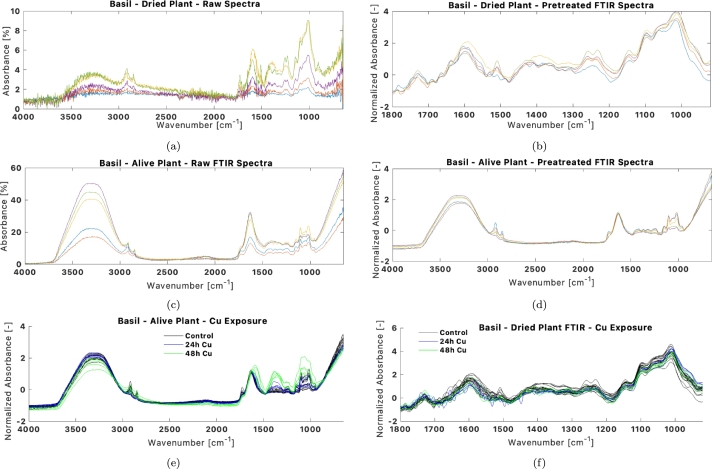
FTIR spectral pretreatment of Basil plant: (a) original FTIR reflectance spectra of the dried healthy leaf; (b) Smoothed, normalized, ROI-cropped spectra of a dried leaf; (c) Original reflectance FTIR spectra of an alive leaf; (d) Smoothed and normalized spectra of an alive leaf. (b) and (c) are the spectral products utilized in PCA modelling. (e) and (f) showcase the alive and respectively dried plant FTIR processed spectra for the *Cu*^2+^ exposure.

The FTIR measurements of the dried leaf, as observed in Fig. 8a, contain much more noise than the alive leaf FTIR or the Raman measurements. The spectra were cut and transformed from reflectance to absorbance by flipping the spectra. The smoothing and normalization made the peaks overlap in a pattern common to each experiment. For the case of the FTIR measurements on alive leaves, the signal-to-noise ratio was very satisfactory, and all spectra could be used in the analysis.

### 4.3 Scenario A: heterogeneity, model calibration and variation studies

**Figure 9:**
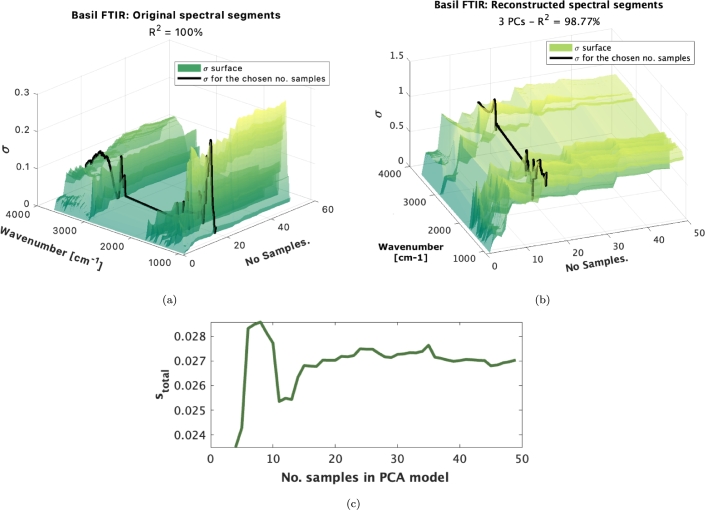
(a) Variation of the dataset as a function of the number of random samples included (b) and variation of the re-projected dataset as a function of the number of random samples included. The black line represents the number of samples chosen in the model; (c) variogram in the PCA model with three principal components.

One possible way to determine the amount of necessary random samples from a healthy plant to calibrate a healthy-plant model is to use a variogram-based tool. Since PCA is already capable of filtering out un-systematic variation, the selection of the number of samples can be based on the re-constructed spectra with the chosen number of principal components utilized in the model. As it can be observed in Fig. 9a, when all the variation is included, the variation of the dataset increases continuously as more samples are added. Some variation is due to noise and interference. When considering only variation captured by the PCA model (Fig. 9b), we can observe a steady small increase in variation after 15 samples. The same is confirmed when looking at the variogram in Fig. 9c.

**Figure 10:**
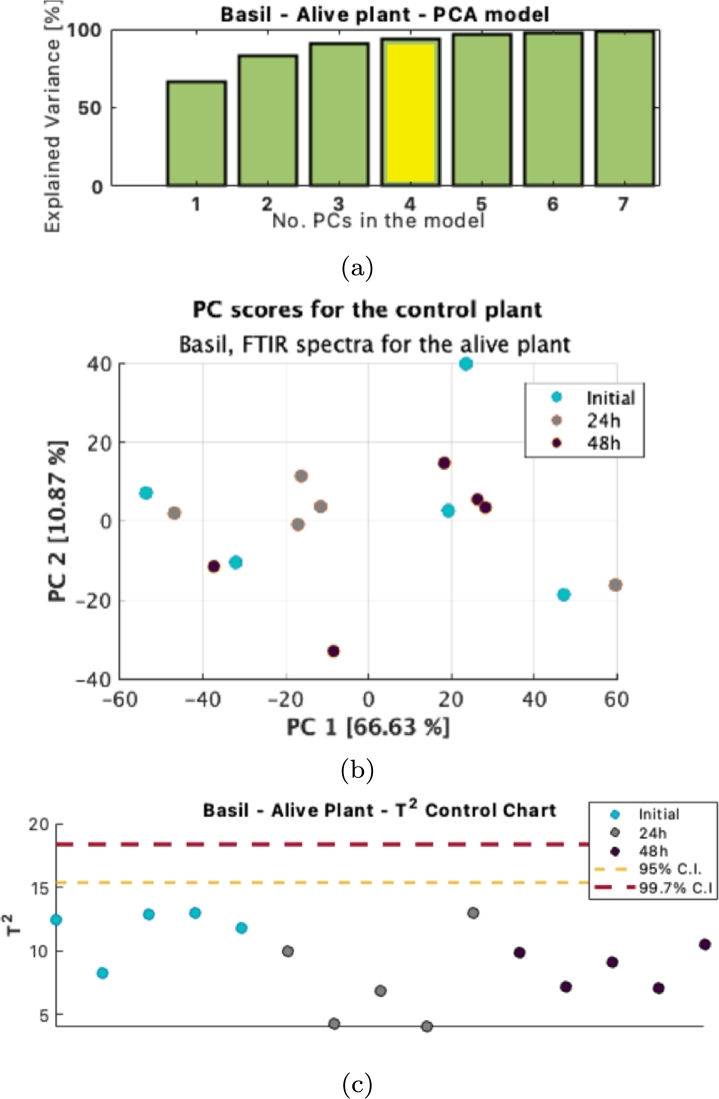
Heterogeneity testing and effect of experimental setup on the Basil Alive Control plant. (a) Explained variance by the model (b) Scatter plot of the first PCs scores (c) T2 control chart for the 4 PCs control model.

To ensure variational differences represent only pollutant-derived changes, three experimental set-ups have been included in the calibration of the “healthy” PCA model: the plant at the start of the experiments, the control plant after 24 h in water, and the control plant after 48 h in water. As seen in Figs. 10b, 10c, the observations cannot be clustered based on their experimental setups. The first principal components showcase that the plant is homogeneous, and no major spectral differences are observed in the variation left unmodeled. Also, no clustering could be observed based on the sampling location on the plant (leaf stem, leaf extremities, old or new leaf).

### 4.4 Pollutant identification

**Figure 11:**
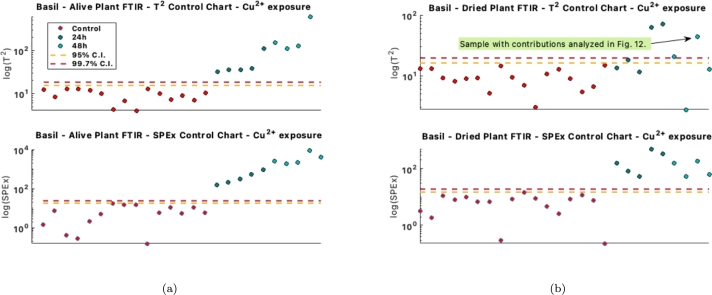
Control charts for identifying polluted samples, in the case of *Cu*^2+^ solution exposure, measured with FTIR in the alive state (a) and in dried samples (b). The scale of the plots is logarithmic due to the large out-of-control values.

After calibrating the model with spectra from the control (healthy) plants, the spectra from the polluted samples are then projected onto control charts. These charts are an effective means to monitor the effects of pollutants on systematic changes in the spectra. This is evident in Fig. 11a, where both the model-captured variation control charts ($T^{2}$ charts) and the charts for new or residual variation (SPEx charts) successfully identify the polluted samples. Notably, as the exposure duration to the pollutant increases from 24 hours to 48 hours, the values in both SPEx and $T^{2}$ control charts rise correspondingly. However, this trend is not observed in the control charts for the dried plants. In this case, the $T^{2}$ charts failed to detect the polluted samples beyond the 98% limit, as illustrated in Fig. 11b.

**Figure 12:**
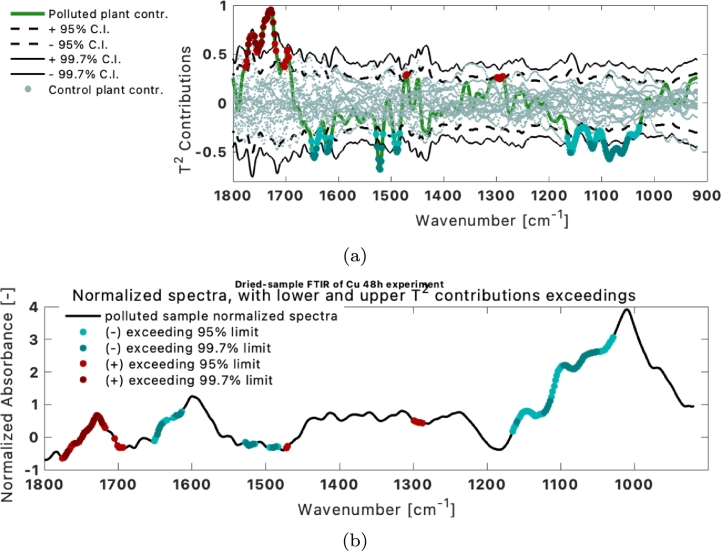
(a) Contributions to the *T*^2^ control chart of individual wavenumbers (b) wavenumbers with exceeding contributions on the normalized spectra.

A further analysis can be taken to attribute high control chart values to variational differences. The contribution of variables (here wavenumbers) to the values in the control charts reveals if the measurement has higher or lower absorbance in a certain wavenumber. For the marked sample in Fig. 11b, the contributions of different wavenumbers to the control charts are presented in Fig. 12a. Fig. 12b showcases that the reason for the exceeding control $T^{2}$ values are: peaks disappearing or lowering intensity (i.e. at 1170 cm^−1^) and other peaks appearing or increasing in intensity (i.e. at 1740 cm^−1^).

A detailed table with the findings is found in Table 2. A notable observation is the consistency in the percentage of samples that present the effect. For example, the effects generated by the Fe exposure are seen in the entirety of FTIR samples, either dry or alive.

**Table 2:** Wavenumbers exceeding the highest control limits (C.I. 99.7%), as per the methodology proposed in **Scenario A**. The methods are (1) FTIR on dried leaf, (2) FTIR on alive leaf, (3) Raman on alive leaf. The *T*^2^↑ [%] and *SPEx*↑ [%] columns count the percentage of polluted samples signalling over the control charts' control limits. The *T*^2^↑ samples present changes in the modelled variation, whereas the *SPEx*↑ samples present new variation that has not been modelled in the healthy plant PCA model.

		Meth.	*T*^2^↑ [%]	*SPEx*↑ [%]	Common *T*^2^ and *SPE*_*x*_ peaks over C.I. 99.8% [cm^−^1]	Comments. Effect in..
**Basil**	**Cu**	(1)	55	100	1487 ↓, 1614(wide) ↓	55% of the samples, representing the 48 h batch.
		(2)	100	100	1543 ↑, 1628 ↑, 1759 ↓, 2957 ↑	60% of the samples.
		(3)	100	100	784 ↑, 840 ↑ 1228 ↓	50-50% of the samples.
	**Fe**	(1)	100	100	1004 ↓, 1101 ↓, 1306 ↑, 1498 ↑, 1585 ↓, 1666 ↑	100% of the samples.
		(2)	100	100	1388 ↑, 1567 ↑, 1647 ↑, 1759 ↓	100% of the samples.
		(3)	90	100	1244 ↑, 1300 ↑, 1612 ↑	30-38% of the samples.
	**Zn**	(1)	26	100	1252 ↑, 1442 ↑	26% of the samples, representing half of the 48 h batch.
		(2)	50	100	1509 ↑	50% of the samples.
		(3)	62	100	1620(wide) ↑, 1244 ↑	23% of the cases.

**Mint**	**Cu**	(1)	40	100	1703 ↑, 1794 ↑	40% of the samples, representing most of the 48 h batch.
		(2)	50	100	1520 ↑, 1628 ↑, 2994 ↓	30-40% of the samples, representing most of the 48 h batch.
		(3)	100	100	732 ↑, 1328 ↑	71.42% of the samples.
	**Fe**	(1)	20	90	1708(wide) ↑	100% of the samples.
		(2)	30	100	1517 ↑, 1626 ↑	100% of the samples.
		(3)	64	100	740 ↑, 852 ↑, 1324 ↑	25% of the samples, representing half of the 48 h batch.
	**Zn**	(1)	90	100	1354(wide) ↓, 1470 ↑, 1694 ↑	70-90% of the samples.
		(2)	50	100	1351 ↓, 1494 ↑, 3017 ↑, 3587(wide) ↓	50% of the samples, representing the 48 h batch.
		(3)	25	100	736 ↑, 1324 ↓	14.26% of the samples.

The $T^{2}$ control charts show if there are changes in the original variational profiles captured in the PCA model. For example, in a healthy plant, the wavenumber at 1580 cm^−1^ negatively correlates with the wavenumber at 1000 cm^−1^ with a certain proportionality coefficient: when the wavenumber at 1580 cm^−1^ increases with one unit, the wavenumber at 1000 cm^−1^ decreases with two units. This can be seen from the healthy-plant PCA model. When a polluted plant spectrum is projected onto the PCA model, if there are changes to the existing variational profiles, it is reflected as an out-of-control point in the control chart. This happens if, for example, the wavenumber at 1000 cm^−1^ decreases by three units instead of two relative to the one-unit increase in the wavenumber at 1580 cm^−1^. In this case, the contributions of the 1000 cm^−1^ wavenumber to the control chart will be highly negative, showing the value is lower than expected in that wavenumber.

The SPEx chart shows if there are new variational profiles present in the data. For example, in the original PCA model, the wavenumber around 1300 cm^−1^ had small loadings in every significant principal component. That means that regardless of the measurement point on the healthy leaf, the wavenumber at 1300 was not changing, or there was no systematic change in the dataset involving that wavenumber. However, after pollution, the wavenumber at 1300 cm^−1^ started to have a strong positive correlation with the wavenumber at 1580 cm^−1^. That is new variation that was introduced into the model, and it will show up as over-the-control points for the SPEx control charts.

While the dried plant altering mechanisms are different, some of the modifications in the living plant can be explained through the different mechanisms by which the plant is affected.

In the basil plant, Cu seems to affect the plant by protein alterations and lipid modifications. One of the protein modifications is seen in the increase at wavenumbers 1543 cm^−1^, which is an indicator for an amide II band found in proteins [43]. An increase in absorbance in this area can suggest either the binding of $Cu^{2+}$ ions to proteins within the plant tissue or an increase in protein content or changes in protein structure as a response to stress. Another protein modification is the increase at 1628 cm^−1^, which indicates the amide I band of proteins, primarily C=O stretching vibrations. It can also overlap with absorbed water's bending vibrations. An increase here could indicate alterations in protein secondary structures, such as an *β*-sheet content, or it might reflect changes in the plant's water content or the structure of water within the plant tissues due to the interaction with $Cu^{2+}$ ions. The decrease at wavenumber 1759 cm^−1^ can be associated with ester linkages in lipids [44] and might suggest a breakdown or modification of lipids within the plant, possibly due to oxidative stress caused by $Cu^{2+}$ ions or alterations in the plant's metabolism in response to Cu exposure.

In the mint plant, we can observe an increase in the wavenumber at 1520 cm^−1^ for the same Cu exposure. The region is typically associated with the N-H bend in the amide II band of proteins or with vibrational modes of nitrogenous compounds [45]. An increase in this band might be more likely related to protein content changes, possibly indicating stress responses that involve alterations in protein structure. It could also affect an increase in certain secondary metabolites related to plant defence mechanisms. An increase at 1628 cm^−1^ might be associated with the amide I band of the proteins due to C=O stretching vibrations within the protein backbone. As in the basil plant, this effect can also be due to increased levels of bound water or changes in the internal water structure, particularly under stress conditions that affect the plant's hydration state [46]. The decrease at 2994 cm^−1^ is most likely related to the C-H stretching vibrations found in aliphatic hydrocarbons, typically in lipids. A decrease in this region could indicate a reduction in the lipid content, an effect observed in the case of Mint as well, and might be due to oxidative stress caused by the $Cu^{2+}$ ion exposure.

### 4.5 Scenario B: mean spectral difference and loadings difference

**Figure 13:**
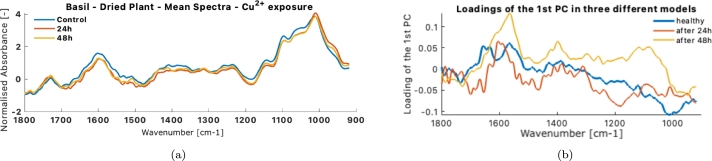
(a) Mean FTIR spectra differences between the healthy plant and the polluted one with Cu in the region of interest of the dried basil plant.

In the lack of a statistical tool to evaluate the spectral differences, spectral differences are observed as a difference of spectra means between the experiments and the healthy plant. From Fig. 13a, that represents the mean spectral differences. It can be observed that the peak around 1740 cm^−1^ maintains its magnitude, whereas the one around 1000 cm^−1^ increases in magnitude. The peak at 1600 cm^−1^ sees a decrease. It is generally difficult to interpret systematic changes when only looking at mean spectra.

To discern spectral differences, one approach involves comparing the loading differences between two distinct models: one calibrated using spectra from healthy plants and the other using spectra from polluted plants (**Scenario B**), as depicted in 13b. A key distinction between **Scenario A** and **Scenario B** lies in the required sample size for accurate analysis. In **Scenario A**, projecting even a single sample from the polluted plant onto the control chart suffices to identify deviations from control samples. However, in **Scenario B**, comprehensive variation studies must be replicated for the polluted plant. The number of samples needed to calibrate a model for a polluted plant accurately may exceed that for a healthy plant. This is due to the uneven impact of the contaminant across different areas of the leaf. Simply selecting a subset of samples from the healthy plant can lead to variable PC loadings. Therefore, without conducting variographic studies on the polluted plant, relying solely on loading differences can be misleading.

### 4.6 Digital imaging: understanding heterogeneous pollution effects

Digital imaging is a useful complement tool to portable spectrometers. As different chemical or physical stress factors can result in the same colour modification in the plant, the tool does not come to perform qualitative analysis but to help in analysis using other instruments.

**Figure 14:**
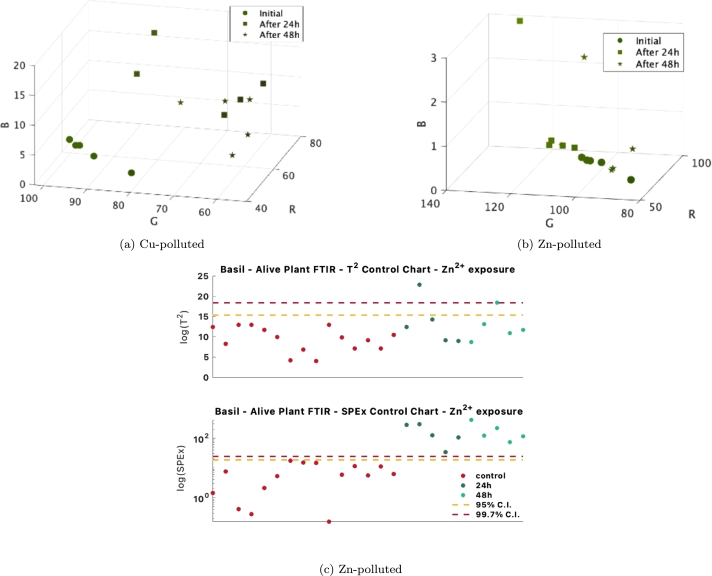
Digital image averages of individual leaves for the (a) *Cu*^2+^ solution experiment and (b) *Zn*^2+^ solution experiment. The *T*^2^ control chart (c) represents the alive-plant FTIR model for *Zn*^2+^ solution exposure.

As it can be observed in Fig. 14a, the colour differences between healthy and polluted samples are clear in the cases in which the control charts also distinguish between the healthy and polluted samples (11a). In other cases, the control charts present a few points above the control limit. This is also the case for the $T^{2}$ control chart in Fig. 14c, where a single point exceeds the control limit, and the polluted samples are not very different from the control, healthy plant. Here, the colourimetric difference between the leaves is handy (Fig. 14b, to provide extra insight, showing that some leaves are more affected than others, thus the difference in control chart values. This is the case in which the surface of the leaf is no longer homogeneous, and discolourations appear on the leaf surface due to exposure to the pollutant in a non-consistent way throughout the plant. Portable spectrometers are more prone to noisy measurements, and digital imaging, in this case, confirmed that the over-control-limits data point originates from a flaw in the plant and not instrumental measurement errors. Fig. 14b reveals that the outstanding points in the Control Chart originate from discoloured spots that are brighter. Thus, they come from effects on the plant surface and are not instrumental inconsistencies. Also, the presence of experiment samples out of control limits in the SPEx control chart (Fig. 14c) can be seen in the tendency to shift towards yellow.

### 4.7 Usage of portable methodologies, limitations and capabilities

A summary of the findings on the usability of the portable methods is found in Table 3. Alive plant FTIR has the clearest spectra, able to distinguish between a healthy and a polluted sample not only qualitatively but also based on the exposure time to the pollutant. Because the point-and-shoot modality of the Raman spectrometer does not perform well when aiming at a leaf attached to the plant, multiple leaves must be stacked together for the focal point to be inside the sample, and it is difficult to assess which point spectra were taken.

**Table 3:** Advantages and challenges of portable instrumentation in pollutant identification.

Method	Sampling from	Advantages	Challenges
Handheld FTIR	Dried plant	The plant is not being destructed by the method.	The signal-to-noise ratio is very high at most parts of the spectrum. The spectrum needs to be cropped at the region of interest to limit the effects.

	Alive plant	The spectrum is very clear, with a good signal-to-noise ratio throughout all wavenumbers reflected. The sample is more homogeneous when measured alive.	The method destroys the plant. Background calibration needs to be renewed after each measurement.

Handheld Raman	Dried plant	The analysis could not be achieved as the laser burns the sample at any intensity available in the tested instrument.	

	Alive plant	The plant is not being destroyed by this method.	The focal point is difficult to measure and set up. Aiming at one plant leaf with the spectrometer and getting a clear spectrum is nearly impossible. To overcome the limitation, multiple leaves need to be under the laser.

	Solution samples	The analysis could not be achieved due to the low signal-to-noise ratios. The concentrations of the solutions are too small to be detected.	

Digital imaging	Alive plant	The plant is not being destructed by the method. Aid in identifying heterogeneous contaminant effects.	The uneven surface of the leaf makes it have darker-coloured areas that do not originate from the leaf itself but represent shadows. Diffuse light must be utilised to overcome the limitation, and the leaves need to be straightened up.

In digital imaging, the colour correction step helps eliminate inconsistencies based on the light intensity. However, the uneven surface of the leaf still presents a challenge to the digital image. For example, the shadowy parts of the mint leaf created more dark spots in the digital image compared to the relative flatness of the basil leaf, shifting the average colour of bigger leaves.

Compared to literature reference review studies on plant disease detection from leaf-spectra [12], [13], [7], [14], the following comparisons can be drawn:

• The present study introduces the effect of metal contaminants as opposed to known plant diseases, a less-researched sub-field.
• The demonstrated method can identify unhealthy plant behaviour as over-the-control spectral points in control charts before prior knowledge of the contaminant, making it applicable to identifying any type of contaminant that poses a risk to the plant's health. In the literature, samples of known diseases are needed prior to model training/calibration.
• For some of the plants that were systematically affected (e.g. Cu^2+^ exposure, alive plant), the method has proven effective in distinguishing between exposure times. These qualities are not achievable with the classification-based mentioned in the reviewed literature.
• The present model is fully interpretable: apart from the discrimination between healthy and polluted states, individual wavenumbers responsible for signalling the structural changes can be analyzed through the model. In the reviewed literature, with the exception of PLS-DA, most of the utilized models were closed and non-interpretable, and a separate analysis needs to be carried out to identify wavenumber dynamics.

## 5 Conclusion

The portable FTIR and Raman instruments have been proven to be efficient in identifying pollutants in food plants. However, measurements from these instruments are more prone to noise than those from their bench-top alternatives. Thus, pretreatment of the spectra, such as region of interest cropping, background correction, and normalization, has to be applied prior to modelling.

Statistical methods that can extract variational profiles and reconstruct the data without noise are suitable for modelling. There exist tools that can distinguish between polluted and healthy samples, such as statistical process control charts ($T^{2}$ and SPEx) derived from principal component analysis. The contribution of wavenumbers to the control chart values gave insights into wavenumbers in which the absorbance was increased or decreased compared to the healthy plant.

These differences in the healthy plant should not be thought of as spectrum-to-spectrum differences but as changes in the systematic variational profile of the leaf. Naturally, the leaf surface is homogeneous and contains a certain amount of variance. If there are pollutant effects on the leaf, either on the leaf as a whole or in regions of the leaf, these changes will be seen as new variation, different than the one that was initially modelled.

In general, clustering methods can be applied to principal component scores to distinguish between healthy and polluted observations, but the exposure times cannot be estimated from the spectra alone. In the case of alive-plant experiments, the exposure time was also determined based on control charts in the case of $Fe^{2+}$ exposure and $Cu^{2+}$ exposure, but could not be determined in the case of $Zn^{2+}$ exposure.

Digital imaging is an inexpensive addition to handheld instruments that allows evaluating whether extreme values in newly acquired spectra are derived from the heterogeneity of the stressed plant or instrumental error.

The present methodology is not limited to plant pollutant identification but can also be utilized in fields where a heterogeneous medium is partially influenced by an external stressor, such as a chemical stressor, for example in rock weathering.

## CRediT authorship contribution statement

**Zina-Sabrina Duma:** Writing – original draft, Visualization, Validation, Software, Methodology, Investigation, Formal analysis, Data curation, Conceptualization. **Tuomas Sihvonen:** Writing – original draft, Investigation, Conceptualization. **Erik Vartiainen:** Validation, Supervision, Conceptualization. **Satu-Pia Reinikainen:** Supervision, Resources, Project administration, Methodology, Formal analysis, Conceptualization.

## Declaration of Competing Interest

The authors declare that they have no known competing financial interests or personal relationships that could have appeared to influence the work reported in this paper.

## Data Availability

Data will be made available by request.
